# Predictive Model of Spread of Progressive Supranuclear Palsy Using Directional Network Diffusion

**DOI:** 10.3389/fneur.2017.00692

**Published:** 2017-12-21

**Authors:** Sneha Pandya, Chris Mezias, Ashish Raj

**Affiliations:** ^1^Department of Radiology, Weill Cornell Medicine, New York, NY, United States; ^2^Department of Neuroscience, Weill Cornell Medicine, New York, NY, United States

**Keywords:** tauopathies, progressive supranuclear palspy, network diffusion, directionality, 4R

## Abstract

Several neurodegenerative disorders including Alzheimer’s disease (AD), frontotemporal dementia (FTD), Parkinson’s disease (PD), amyotrophic lateral sclerosis, and Huntington’s disease report aggregation and transmission of pathogenic proteins between cells. The topography of these diseases in the human brain also, therefore, displays a well-characterized and stereotyped regional pattern, and a stereotyped progression over time. This is most commonly true for AD and other dementias characterized by hallmark misfolded tau or alpha-synuclein pathology. Both tau and synuclein appear to propagate within brain circuits using a shared mechanism. The most canonical synucleopathy is PD; however, much less studied is a rare disorder called progressive supranuclear palsy (PSP). The hallmark pathology and atrophy in PSP are, therefore, also highly stereotyped: initially appearing in the striatum, followed by its neighbors and connected cortical areas. In this study, we explore two mechanistic aspects hitherto unknown about the canonical network diffusion model (NDM) of spread: (a) whether the NDM can apply to other common degenerative pathologies, specifically PSP, and (b) whether the directionality of spread is important in explaining empirical data. Our results on PSP reveal two important findings: first, that PSP is amenable to the connectome-based ND modeling in the same way as previously applied to AD and FTD and, second, that the NDM fit with empirical data are significantly enhanced by using the directional rather than the non-directional form of the human connectome. Specifically, we show that both the anterograde model of transmission (some to axonal terminal) and retrograde mode explain PSP topography more accurately than non-directional transmission. Collectively, these data show that the intrinsic architecture of the structural network mediates disease spread in PSP, most likely *via* a process of trans-neuronal transmission. These intriguing results have several ramifications for future studies.

## Introduction

Several neurodegenerative disorders, including Alzheimer’s disease (AD), frontotemporal dementia (FTD), Parkinson’s disease (PD), amyotrophic lateral sclerosis (ALS), and Huntington’s disease (HD), report aggregation and transmission of pathogenic proteins between cells (1–6). The topography of these diseases in the human brain also, therefore, displays a well-characterized and stereotyped regional pattern and a stereotyped progression over time. This is most commonly true of AD, as well as other dementias characterized by hallmark misfolded tau or alpha-synuclein pathology, both of which appear to propagate within brain circuits using a shared mechanism. The most canonical tauopathy is AD, but a diverse group of related taupathies are known: FTD, corticobasal degeneration, semantic dementia, and many more (7). Far less studied is a rare disorder called progressive supranuclear palsy (PSP). The hallmark pathology and atrophy in PSP are similar to those in other tauopathies, and its regional patterning is likewise highly stereotyped: initially appearing in the striatum, followed by its neighbors and connected cortical areas (8, 9).

The mechanisms underlying stereotyped patterning and progression in tauopathies are not fully understood, and both cell–cell communications governed by anatomical and functional connections, and cell autonomous molecular factors characterized by gene expression signatures, could conceivably play a role in vulnerability to disease spread (10–15). Foremost among non-cell autonomous factors is network connectivity, which is increasingly considered a plausible and even key driver of vulnerability (16–18).

Assuming that trans-neuronal transmission must proceed along axonal projections, the spatiotemporal dynamics of pathology spread can be given quantitatively and deterministically from the inter-regional anatomic connectivity patterns (19). Modern diffusion-weighted magnetic resonance imaging (dMRI) (20) and post-processing techniques like fiber tractography (21) and connectivity mapping (22) have enabled the computation of inter-regional connectivity, yielding what is frequently called the human “connectome.” Recently, our group proposed a mathematical model of prion-like trans-neuronal spread of neurodegenerative pathology called the network diffusion model (NDM), evolved on dMRI-based structural networks or connectomes. This model demonstrated that observed spatial patterns of neurodegeneration in common degenerative diseases like AD and FTD can be explained simply as a consequence of network spread (16). This model also gives an explanation of selective regional vulnerability in terms of disease epicenters, called eigenmodes, associated with pathology. Since the model is deterministic, it was successfully employed to predict future atrophy patterns of AD subjects from their baseline patterns and connectomes (17).

In this study, we explore two mechanistic aspects hitherto unknown about the canonical NDM of spread: (a) whether the NDM can apply to other common degenerative pathologies, specifically PSP, and (b) whether the directionality of spread is important in explaining empirical data. We, therefore, apply the NDM to quantitatively assess whether trans-neuronal transmission of PSP pathology can recapitulate observed PSP topography. Our empirical data come from an unprecedented dataset of 60 PSP subjects from the 4-Repeat Tauopathy Neuroimaging Initiative (4RTNI) study, a multinational prospective observation study that examines clinical, radiologic, and biological findings of disease progression in tauopathic individuals, including PSP.^1^ Our interest in PSP arises from its distinct spatial pattern in comparison to AD; hence the ability of NDM to explain PSP pattern would contribute to the emerging notion that all neurodegenerative pathologies follow shared mechanisms of spread.

To assess the second question, we propose a novel construction of a *directional human connectome*, for the first time. Clearly, directionality of tracts or inter-regional anatomic connectivity, as defined by the polarity of individual axonal projects (soma to axonal terminal or *vice versa*) is impossible to determine from dMRI data, as water diffusion along fiber bundles does not respect cell polarity. Instead, we exploit the well-known fact that homologous structures exist between many species, for some of whom we do happen to have anatomic connectivity data from painstaking tracer studies. Using retrograde tracer studies, a detailed mesoscale mouse connectome was reported by the Allen Institute (23). We, therefore, developed a novel technique whereby human and mouse brain atlas parcellations are used to define homologous brain structures, and the Allen mouse directional connectivity is transferred to non-directional human connectome. The importance of studying directional connectomes is that *in vitro* and *in vivo* mouse studies are increasingly revealing directional preference in the transmission of various misfolded proteins; however, conclusive data on directionality of each protein are not currently established (24).

Our results on PSP reveal two important findings. First, that PSP is amenable to the connectome-based ND modeling in the same way as previously applied to AD and FTD. This establishes that PSP might propagate using the anatomic connectome in the same way that is known for AD and FTD. Second, we found that the NDM fit with empirical data are enhanced by using the directional rather than the non-directional form of the human connectome. Specifically, we show that the anterograde model of transmission (some to axonal terminal) explains certain aspects of PSP topography more accurately than the non-directional model. Similarly, certain aspects of PSP are better explained by the retrograde mode of transmission. Overall, both directional models outperform the non-directional model, and retrograde mode is overall the most accurate. These intriguing results have several ramifications for future studies.

## Materials and Methods

### Building an Anatomic Connectome from Parcellated Atlas and dMRI of Healthy Cohort

Axial T1-weighted structural fast spoiled gradient-echo scans (TE = 1.5 ms, TR = 6.3 ms, TI = 400 ms, 15° flip angle, 230 × 230 × 156 isotropic 1 mm voxels) and high angular resolution diffusion imaging data (55 directions, *b* = 1,000 s/mm^2^, 72 1.8-mm-thick interleaved slices, 0.8594 mm × 0.8594 mm planar resolution) were acquired on a 3T GE Signa EXCITE scanner from 73 fully consented young healthy volunteers under a previous study approved by Weill Cornell’s institutional review board; for details of study protocols see Ref. (25). The exclusion criteria were pregnancy, a history of neurological or psychiatric diagnosis, seizure, or drug or alcohol abuse. Demographic characteristics of these young healthy subjects are shown in Table 1. Tractograms were extracted from these 73 young healthy subjects to create the normative connectome for the study. The measure of connectivity used in this paper is the anatomical connection strength (ACS) as proposed in Ref. (26). The overall differences in regional connectivity were normalized by a scaling factor equal to the sum of the connections. Thus, the connection strength, *c_i,j_*, between ROIs *i* and *j*, was defined as the ACS score of streamlines connecting the two regions *i* and *j*. We refer to this network as graph *G* = {*V*,*E*} whose nodes ν*_I_* ∈ *V* represent the ith GM region, and edges *e_i,j_* ∈ *E* represent white matter fiber pathways whose connection strength is *c_i,j_*. Connections are assumed to be bidirectional since directionality is not deducible from diffusion tensor imaging (DTI) tractography data. Gray matter regions from the T1-weighted images were parcellated using FreeSurfer volumetric pipeline (27) and a Desikan-Killiany atlas (28) into 68 cortical regions, 34 from each hemisphere and 18 subcortical regions. Six subjects were eliminated from the 73-subject dataset due to FreeSurfer failure or insufficient MR contrast.

**Table 1:** Demographic and clinical details of young healthy controls used to construct canonical structural connectome, PSP, and age-matched healthy cohort.

	Male	Female	Age	MoCA	UPDRS-II
Young HC (*n* = 73)	40	33	30.2 ± 6.7	–	–
PSP (*n* = 65)	29	36	70.5 ± 7.4	24.8 ± 5.1	33.2 ± 17.2
Age-matched HC (*n* = 150)	75	75	75.435 ± 6.7	–	–

*MoCA, Montreal Cognitive Assesement; UPDRS, Uniform Parkinson’s disease Rating Score*.

### Obtaining Directional Human Connectomes by Utilizing Directional Information from Mesoscale Mouse Connectomic Data

#### The Allen Brain Institute (ABI) Mouse Connectivity Atlas

Connectivity data were taken from the supplementary dataset published along with the mesoscale mouse connectome from the ABI (23). The ABI generated their mouse connectivity data using an anterograde viral tracer engineered to express GFP natively using a promoter with high affinity for the ubiquitous transcription factor synapsin (23). The specific vector they used, rAAV1, travels exclusively along axons, across synapses, and through dendrites in the CNS, and almost exclusively travels in an anterograde direction (over 99% of the time). Viral vectors were injected and then mice were sacked after a period of approximately 3 weeks and their brains assessed for the expansion of GFP expressing viruses into other regions and for expansion of the GFP expressing virus within the targeted region. Brains were then sliced and each slice was analyzed by an automated camera system to sum the number of pixels across all voxels in each cytoarchitectonically defined brain region; these values were then normalized by injection volume, to give a per voxel connectivity density measurement between any two areas out of the total 213 regions defined in the data. The cohort of mice used to generate the dataset was of *N* = 1,231 C57/BL6 males, and regions were taken from the previously created ABI Mouse Brain Ontological Reference Atlas, created using specific staining for regional cell types with a cohort of *N* = 469 C57/BL6 male mice. C57BL/6 male mice are a common inbred strain of laboratory mouse and the most widely used mice for use as models of human disease and neurodegenerative disorders. There are several differences in connectivity between mouse lines. No comparable mesoscale whole brain connectome has ever been created for another mouse strain, hence at this point, any large-scale wiring differences in the connectomes of different mouse strains are unknown. Further methodological details and descriptions of the assumptions used to create the dataset can be found in the supplementary information in Ref. (23).

Total projection density between regions was generated by multiplying element-wise by the rows the connectivity matrix times the number of voxels in each seeding region. Next, the 426-region mouse connectome was used to inform the directionality of specific connections in the 86-region human connectome C. Custom MATLAB code then transformed the ABI connectivity matrix C_ABI_ of size 426 × 426, whose nodes reflected a parcellated atlas consisting of 426 GM regions, into a custom connectivity matrix of size 86 × 86, based on the human atlas parcelation. To achieve this, the afferent and effect connections of all samples that fell within the (i,j)-th pair of human homolog regions were summed to give the in- and out-degrees of the human regions: degree_in_ and degree_out_. The overall directionality was estimated by the anterograde ratio $D^{\text{ant}}=\frac{{\text{degree}}_{\text{in}}}{{\text{degree}}_{\text{in}}+{\text{degree}}_{\text{out}}}$. This directionality ratio was then applied to the non-directional human connectome *C* to obtain the (anterograde) directed connectome *C*^ant^ = *D*^ant^*.C*. Most of the neocortical connections are bidirectional, and only a small percentage shows strong directionality (see Results). These connections emanate from primary sensorimotor cortices and subcortical structures, as expected. In order to ensure that mouse directionality was relevant to human data, each strongly directional connection was qualitatively verified from macaque data available in the literature, especially from the CoCoMac database (29), which provides an ordinal measure of directionality (one of three levels of connectivity in either direction).

We applied directional connectivity to all human regions for which there were clear mouse analogs: primary sensory neocortical regions, and subcortical regions. The mouse atlas was transferred to a human atlas by taking directional connectivity ratios (outgoing/incoming) per region-to-region connection for all human region pairings both having mouse analogs. We then multiplied the directed connectivity ratios from mouse analog region pairings with the region-to-region connectivity strength in the Desikan Atlas. Here, we define mouse analogs to human regions in two ways: first, we used region pairings where both human regions had directly comparable mouse regions. For example, the precentral gyrus—postcentral gyrus connection is anatomically analogous to primary motor area—primary somatosensory area connection in mice. Second, we combined mouse regions from the Allen Institute atlas into the larger human regions from the Desikan Atlas when the larger combined region in mice was directly analogous to a human region. For instance, the Allen Institute atlas has the hippocampus broken into the various CA regions and the subiculum; the connectivity profiles of these were summed into one hippocampal region, as per the Desikan Atlas, before taking directional connectivity ratios of connections with the hippocampus. All regions not fitting one of the two above mentioned criteria were ignored and no directionality was imputed on human region-to-region connections where both regions did not have a mouse analog. Prior work has noted similarities between mouse and functional connectomes and the regions of the default mode network (30), suggesting that for a first pass at applying directed connectivity to a human connectome, mouse directional connectivity ratios can suffice. However, all results should be interpreted through the lens of the clear limitations of imputing gross wiring patterns from one species to another.

### Age-Matched Normal and PSP Cohorts

Data used in preparation of this article were obtained from the 4RTNI database (see text footnote 1). Neurological and physical examinations, cognitive testing, behavioral testing, and other clinical data from PSP subjects between the ages of 45 and 90 were collected over three longitudinal visits (baseline, 6 months, and 12 months). The conditions for exclusion were any significant neurological disease other than PSP, including PD, multi-infarct dementia, HD, brain tumor, multiple sclerosis, or history of significant head trauma followed by persistent neurological deficits or known structural brain abnormalities. Demographic characteristics of these subjects are shown in Table 1. For our study, we used 60 PSP subjects after elimination due to unknown age, gender, and failed FreeSurfer quality control. Age-matched healthy controls (NC) data used in this study were obtained from the public Alzheimer’s disease neuroimaging initiative (ADNI) database^2^ consisting of multimodal imaging data on AD and healthy subjects. T1-MPRAGE, FLAIR, DTI, ASL-perfusion (ASL), and resting state functional MRI (rs-fMRI) MRI sessions were acquired at 3T. The parameters for the PSP scans were chosen to match those used by the ADNI, including those being developed for ADNI-2 for ASL, rs-fMRI, and DTI at the time. Two-sample *t*-test was used to generate a random sample of 150 age-matched controls from ADNI database. Demographic characteristics of these subjects are shown in Table 1. This randomly generated sample of 150 age-matched healthy controls was used to generate a group atrophy vector for the study.

68 cortical and 18 subcortical volumes from 3T T1-weighted baseline MRI images were extracted using FreeSurfer software for both the cohorts. Estimated Total Intracranial Volume generated by FreeSurfer was used as an estimate for intracranial Volume (ICV) as a normalization measure to correct for head size in this study. ICV has previously been used in several studies for normalization particularly for neurodegenerative disorders for better estimation of regional atrophy that is caused by pathology (31, 32). Image processing steps were visually inspected for white–gray matter boundary and skull-stripping errors to ensure they had been carried out correctly. Subjects were eliminated from the dataset due to FreeSurfer failure or insufficient MR contrast. A vector of regional atrophy was created by using a two tailed *t*-test between PSP and NC mean ICV corrected regional volumes such that *t*_PSP_ = {*t*_PSP_(*i*)|*i* ∈ [1,*N*]} (*N* = 86). The *t*-statistic was converted to the natural range [0,1] using the logistic transform, following (17). These atrophy measures were then used to test the propagation modeling analyses.

### A NDM for Taupathy Spread

We modeled taupathy progression as a diffusion process on graph *G*. From Ref. (16) the transmission of pathology to all brain regions *via* the whole brain disease vector ***x***(*t*) = {*x*(ν,*t*), ν ∈ *V*} and:

(1) $$\frac{dx(t)}{dt}=-\beta Hx(t),$$

where β is a global diffusivity constant and *H* is the well-known graph Laplacian.

$$H=I-D^{-1}C,$$

where *D* is a diagonal matrix whose diagonal entries contain the degree of each node, degree being defined as the sum of weighted connections emanating from the node. Note, in order to accommodate regions having widely different out-degrees, we have used above the degree-normalized version of the Laplacian matrix (17).

#### Directional Laplacian

Next, we define a directional (anterograde) connectome *C*^ant^. Since this matrix is non-symmetric, we define for each node an in-degree and an out-degree given by row and column sums of the matrix: $d_{\text{row},i}=\sum_{j}C_{i,j}^{\text{ant}},d_{\text{col},j}=\sum_{i}C_{i,j}^{\text{ant}}$. Define *H*^ant^ as the anterograde graph Laplacian matrix

$$H^{\text{ant}}=I-diag{\left(d_{\text{row}}.d_{\text{col}}\right)}^{-\frac{1}{2}}C^{\text{ant}}$$

Equation 1 is applied to both the non-directional and anterograde matrices and in each case admits a closed-form solution ***x***(*t*) = *e*^−β^*^Ht^x*_0_, where *x*_0_ is the initial pattern of the disease process, and we call term *e*^−β^*^Ht^* the *diffusion kernel* since it acts essentially as a spatial and temporal blurring operator on *x*_0_. The unit of model’s diffusion time *t* is arbitrary. Global diffusivity β is unknown; hence, we chose a value that would roughly span tau progression (10–20 years), giving β = 0.15. In future, both *t* and β would be estimated by fitting to longitudinal data, and would then acquire correct dimensions and units.

Subsequent diffusion of tau pathology out of potential seed at the *k*-th region is given at any time point *t* by

(2) $$x_{\text{PSP}}(t)=e^{-\text{β}Ht}e_{k},$$

where ***e****_k_* is a unit vector with 1 at the *k*-th entry and 0 elsewhere. Note that although the above model involves pathology, what we have available to us empirically regional MRI-derived atrophy. Hence, an underlying assumption in all analyses henceforth is that the empirical atrophy vector is proportional to the pathology vector, hence both are given by ***x*_PSP_**(*t*).

### Statistical Analysis

Two cerebellar regions were removed, leaving regional PSP atrophy statistics on 84 cerebral regions. The NDM is described by ***x***(*t*) and Φ(*t*), two 84 × 100 tables that represent the pathology (unobserved) and atrophy (measured empirically) in all 84 ROIs over 100 points in time. Pearson correlation strength (*R* statistic) and associated *p*-values were calculated comparing the empirical atrophy vector, *t*_PSP_, with the ND atrophy pattern at all 100 points in time.

#### Repeated Seeding

Next, the NDM was run 84 times, each time starting from a different ROI, in order to deduce the most likely seed regions. For each node *i*, we start the model with *x*_0_ = *e_i_*, where *e_i_* is a unit vector with 1 at the *i*-th location and 0 elsewhere. In the current case, we chose to seed bilaterally, so that two entries in the “unit” vector were assigned 1. This was repeated for each region in turn, and the NDM-predicted atrophy pattern Φ*^i^* (*t*) was calculated from Eq. 3. This gave 84/2 = 42 different Φ (*t*) matrices. For each predictor matrix, the corresponding Pearson’s *R* was calculated at all model times *t*, giving *R^i^* (*t*). These *R^i^* (*t*) values were plotted on common axis, giving what we denote as “*R*–*t* curves.” From each *R^i^* (*t*), we recorded the maximum value $R_{\text{max}}^{i}$, which is used here as an indicator of model evidence reflecting the likelihood of the region i being the true region of pathology onset. This effectively “ran the NDM backwards,” allowing us to determine which seeded ROI would serve as the most likely origination site to subsequently yield the regional patterns closest to empirical data.

#### Potential Seeding

Given that hypothalamus (HTH) seeding produced the best *R* against empirical data (see Results), we explored seeding from these regions, given by the initial vector *x*_0_ = *e_HTH_*, which yields the model predictor Φ*^PSP^*(*t*) = exp(−β*Ht*)*e_HTH_*. Snapshots of the evolving Φ^PSP^(*t*) vector were recorded and plotted in glassbrain renderings at selected model times *t* = 7, 15, 22 years with non-directional connectivity, *t* = 13, 26, 39 years with anterograde directional connectome, and *t* = 14, 27, 41 years with retrograde directional connectome.

#### Random Scrambling

In order to build a null distribution for assigning significance to the NDM, we performed two levels of randomization experiments. (1) We ran the NDM on 2,000 randomly scrambled versions of the connectivity matrix C. C was scrambled using a symmetric transformation of the network’s nodes by randomly permuting entire rows and columns, and the NDM was evaluated for each shuffled network after bilateral HTH seeding for PSP. This scrambling procedure maintains the edge and node statistics of the true connectivity C. The NDM evaluated on these 2,000 randomly scrambled networks, therefore, constitute null or reference models which supplied significance values to results of the true model. (2) We ran the NDM on 2,000 randomly scrambled PSP atrophy vector. Atrophy values in *t*_PSP_ vector were randomly assigned among the 84 cerebral regions with 2,000 different permutations. This scrambling method maintained the true connectivity C, but replaced true regional atrophy pattern with a random distribution of atrophy.

## Results

### Cross-sectional Spatial Distribution of PSP Atrophy

**Figure 1:**
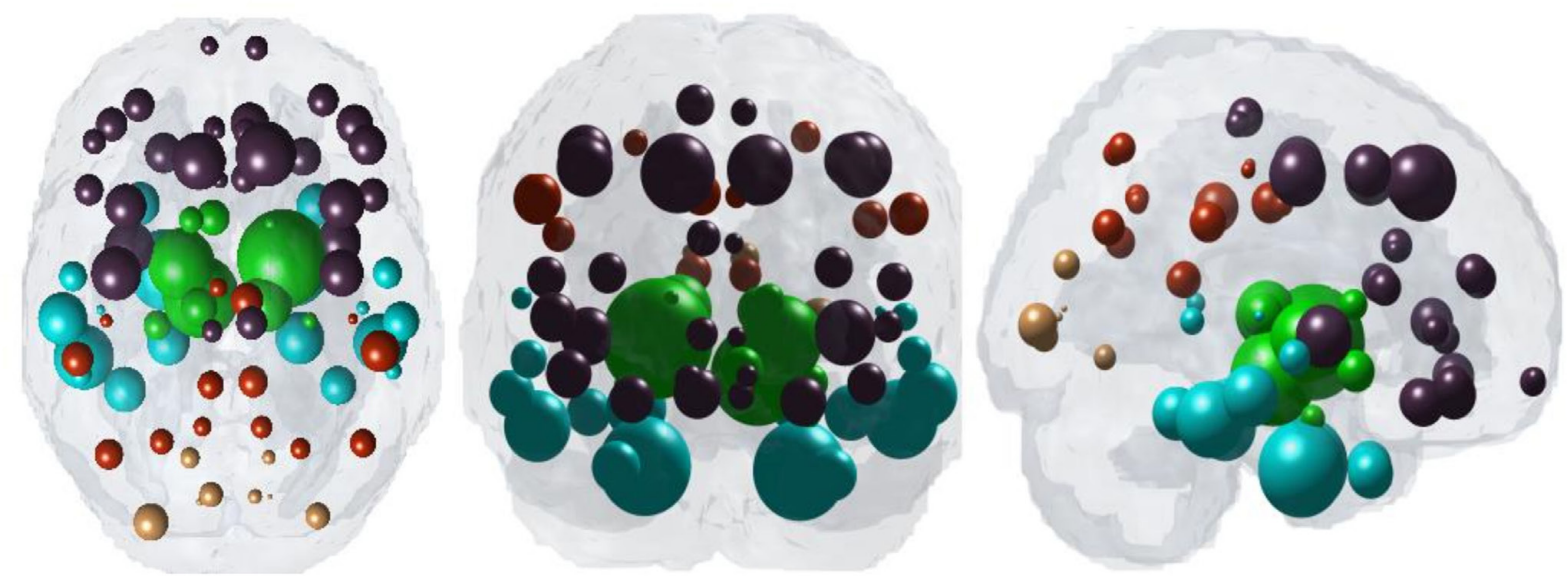
Spatial distribution of PSP atrophy. Measured regional PSP atrophy are depicted by “glass brain” visualization, with spheres placed at the centroid of each brain region, and their diameter proportional to the *t*-statistic of PSP atrophy after logistic transform. Spheres are color coded by lobe—frontal, purple; parietal, red; occipital, orange; temporal, cyan; subcortical, green. Hypothalamus, pallidum, entorhinal, inferiortemporal, and superior frontal regions are top five most atrophied regions as seen by the largest spheres.

Figure 1 illustrates “glass brain” renderings of the spatial distribution of PSP atrophy. The spheres are located at the centroid of each of 84 brain regions, their size is proportional to the *t*-statistic of PSP atrophy after logistic transform and color coded by lobe per: frontal, purple; parietal, red; occipital, orange; temporal, cyan; and subcortical, green. The relative order of regional atrophy in this cohort roughly mirrors the spatial pattern of atrophy. HTH, pallidum, entorhinal, inferiortemporal, and superior frontal are the most atrophied regions as seen by the largest spheres in Figure 1.

### Characterizing the Directional Connectome

**Figure 2:**
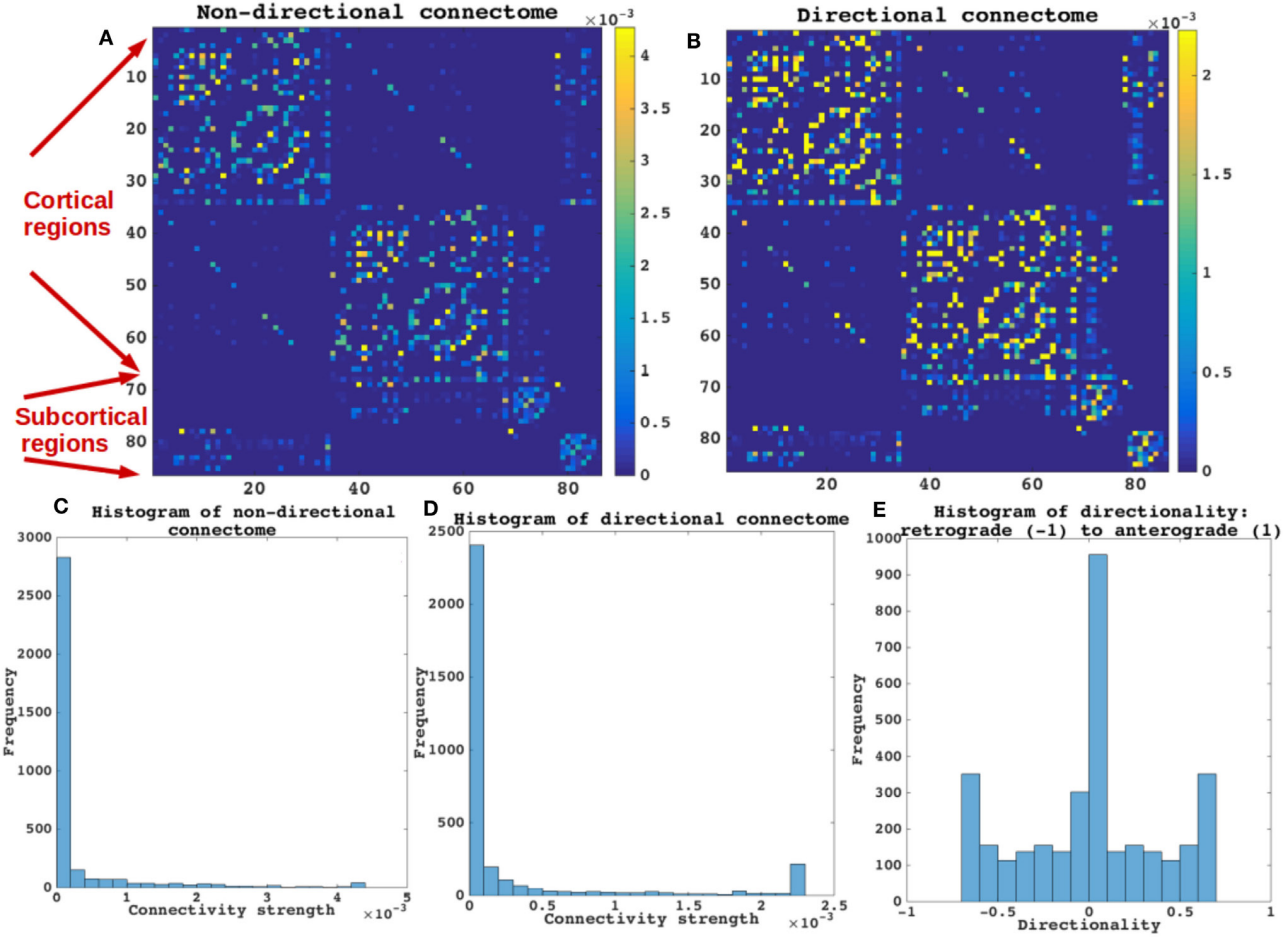
Categorizing non-directional and directional connectome. The properties of the directional connectome built using both human and mouse connectome are described. **(A,B)** The connectivity patterns as seen both in **(A,B)** are very similar. **(C,D)** Connectivity strengths represented by histograms of both the directional and the non-directional connectomes are also very similar. As expected the values of connections are not different between the non-directional and the directional connectome. **(E)** Histogram of the directionality defined by (*C_i,j_* − *C_j,i_*)/(*C_i,j_* + *C_j,i_*), such that −1 represents a purely retrograde connection, and +1 represents a purely anterograde connection. Most connections are bidirectional (value 0) in E and a small minority is directional, with equal numbers in both directions. Mainly subcortical structures display strong directionality [bottom and right portions of the matrices displayed in **(A,B)** while most corticocortical connections are bidirectional].

The properties of the directional connectome built using both human and mouse connectome are described in Figure 2. First, we show that the connectivity patterns (Figures 2A,B) as well as the statistics of both the directional and the original non-directional connectomes are very similar (Figures 2C,D), as they should be. The directionality, defined such that −1 represents a purely retrograde connection, and +1 represents a purely anterograde connection, is shown as a histogram in Figure 2E. Clearly, most connections are bidirectional (value 0) and a small minority is directional, with equal numbers in both directions. Mainly subcortical structures display strong directionality (bottom and right portions of the matrices displayed in Figures 2A,B) while most corticocortical connections are bidirectional.

### Repeated Seeding of the NDM with Non-Directional Connectivity

**Figure 3:**
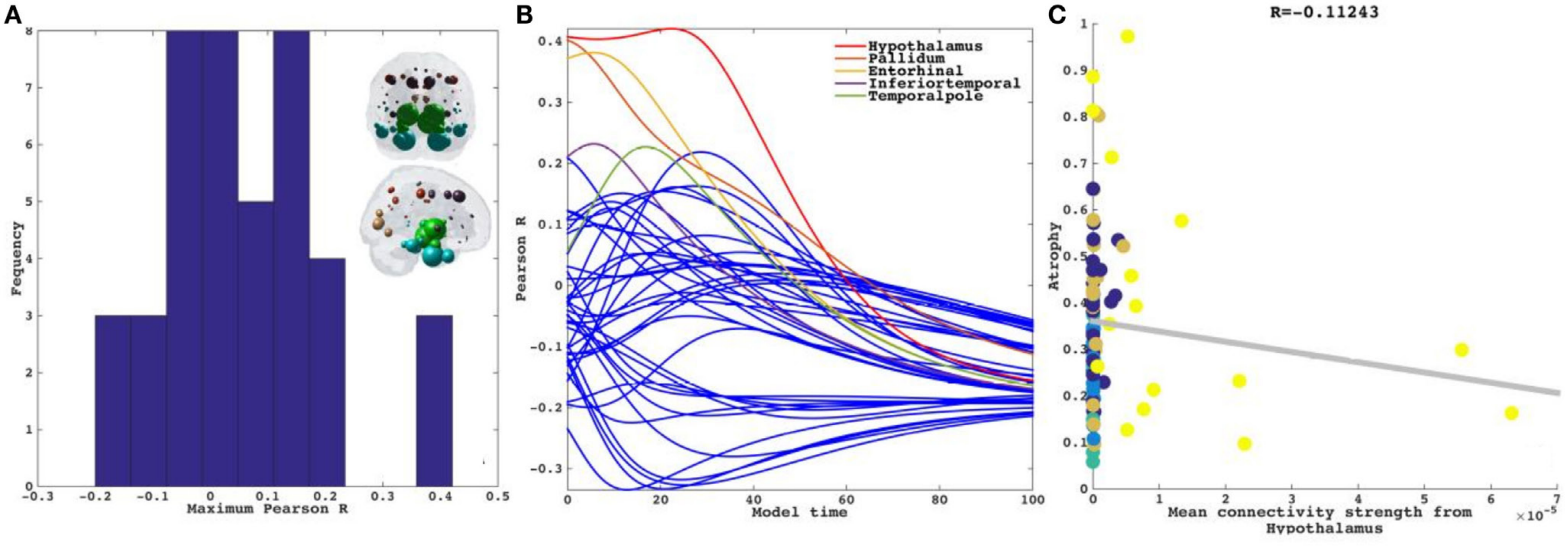
Results for repeated seeding analysis. Each region was seeded in turn and network diffusion model (NDM) was played out for all time points. Pearson’s *R* was recorded at each time point between the model and PSP atrophy vector. As the diffusion time increases, more and more of the pathogenic agent escapes the seed region and enters the rest of the network. **(A)** The point of maximum correlation with measured atrophy using structural connectome was recorded with glass brains of measured *R* shown inset in PSP. **(B)** NDM seeded at bilateral regions using structural connectome indicates that the hypothalamus (HTH) (shown by red curve) could be a plausible candidate for PSP disease seeding—it has the highest peak *R*, and the characteristic intermediate peak indicative of true pathology spread. The unit of model’s diffusion time *t* is arbitrary. For the purpose of demonstration, we consider this arbitrary timescale parameter in terms of “years” but without a robust fitting approach with longitudinal data these time values should be considered only roughly equivalent to years. **(C)** Once best seed was determined we looked at correlations of mean connectivity from HTH versus empirical PSP atrophy. We see a non-significant correlation between PSP atrophy and connectivity from bilateral HTH (*R* = −0.11, *p* < 0.01). Dots are color coded by lobe—frontal, purple; parietal, red; occipital, orange; temporal, cyan; and subcortical, green.

Next each region was computationally “seeded” in turn and NDM was played out over time on the canonical healthy connectome. Figure 3A shows spread of maximum Pearson correlation strength $R_{\text{max}}^{i}$ corresponding to the best fit between empirical data and the NDM seeded at region *i*. The distribution of $R_{\text{max}}^{i}$ is being shown as a histogram. Since $R_{\text{max}}^{i}$ serves as a measure of the likelihood of each region being a seed, this information is spatially depicted in the “glass-brain” insets. Table 2 shows top 20 regions with maximum correlation strength for each region seeded in turn. Clearly, the HTH and other limbic structures serve as the best seed regions for PSP. Figure 3B shows the *R*–*t* curves between the model evolution (Eq. 2), seeded at each region in turn and empirical PSP atrophy. For seed regions that are plausible, this would yield an intermediate peak in *R* where the NDM best matches empirical data, then diffusing uniformly and resembling actual data less and less. The highest *R* was achieved by the HTH, a region known to suffer early atrophy and could most likely be a seed to PSP for the connectome-based network diffusion.

**Table 2:** Top 20 regions with maximum correlation strength for bilaterally seeded ROIs without and with directional connectivity.

Regions from structural bidirectional connectome		Regions from structural anterograde directional connectome		Regions from structural retrograde directional connectome	
Hypothalamus (HTH)	0.42	HTH	0.48	HTH	0.53
Pallidum	0.40	Amygdala	0.44	Amygdala	0.46
Entorhinal	0.38	Entorhinal	0.41	ThalamusProper	0.43
Inferiortemporal	0.23	Hippocampus	0.41	Hippocampus	0.42
Temporalpole	0.23	Pallidum	0.41	Entorhinal	0.41
Amygdala	0.22	ThalamusProper	0.35	Pallidum	0.40
Superiorfrontal	0.21	Putamen	0.31	Temporalpole	0.35
ThalamusProper	0.16	Parahippocampal	0.27	Parahippocampal	0.31
Hippocampus	0.16	Caudate	0.25	Fusiform	0.27
Putamen	0.15	Accumbensarea	0.24	Inferiortemporal	0.27
Parahippocampal	0.15	Temporalpole	0.24	Putamen	0.27
Caudalmiddlefrontal	0.14	Inferiortemporal	0.22	Superiorfrontal	0.21
Fusiform	0.13	Superiorfrontal	0.21	Superiortemporal	0.19
Precentral	0.12	Fusiform	0.21	Insula	0.18
Insula	0.12	Insula	0.18	Middletemporal	0.15
Middletemporal	0.10	Precentral	0.12	Transversetemporal	0.14
Caudate	0.08	Lateralorbitofrontal	0.12	Precentral	0.12
Accumbensarea	0.06	Caudalmiddlefrontal	0.12	Caudate	0.11
Parsopercularis	0.05	Medialorbitofrontal	0.11	Caudalmiddlefrontal	0.11
Rostralmiddlefrontal	0.05	Parstriangularis	0.10	Bankssts	0.10

Next in a model-free analysis, we establish the role played by proximate anatomic features in governing the regional patterns of PSP atrophy. We considered HTH as anatomic predictors based on highest *R* achieved from repeated seeding analysis above. Each covariate is a 84-long vector, covering the entire brain. Since the group atrophy *t*-statistic is generally bilateral, we removed lateralization effects by averaging the left- and right-hemispheric values of these vectors, giving predictor vectors of size 42 × 1. Linear bivariate correlation analyses of regional PSP atrophy with these proximate predictors are shown in Figure 3C. Dots are color coded as per lobes (frontal, purple; parietal, red; occipital, orange; temporal, cyan; and subcortical, green). We see a non-significant negative correlation between PSP atrophy and connectivity from bilateral HTH (*R* = −0.11, *p* < 0.01).

### Network Diffusion Out of Potential Seed Recapitulates Regional Atrophy in PSP Tauopathy Using a Non-directional Connectome

**Figure 4:**
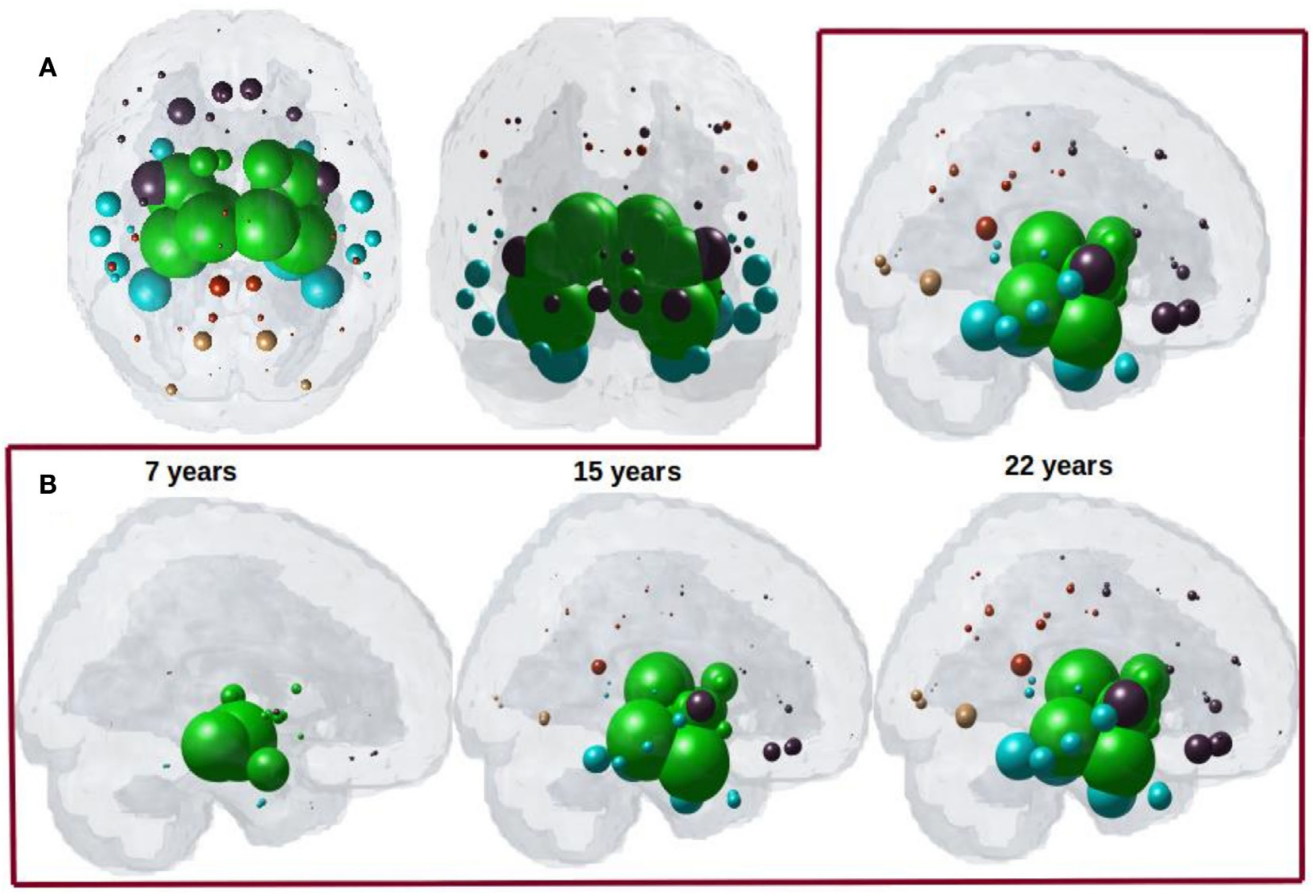
**(A)** ND prediction from bilaterally seeded hypothalamus (HTH) with non-directional connectome. Glass brains of network diffusion model seeded at the bilateral HTH at *t* = 22 years shows spatial pattern from HTH using bidirectional connectome. Subcortical striatal structures especially caudoputamen and globus pallidus, and limbic areas like amygdala, hippocampus, and thalamus which are shown in green are the most affected region. **(B)** Spatiotemporal evolution of empirical PSP pathology. Evolution of HTH seeded network diffusion exhibits the, limbic, striatal, and temporal areas as early affected regions, followed by somewhat slower diffusion into the frontal and parietal regions. This spatial sequencing predicted by the model is a close match with PSP tau progression. We selected three equidistant years at *t* = 7, 15, and 22 years between early stage (*t* = 0) with no diffusion through mature stage (*t* = 22). Here, time is arbitrary, and the use of “years” is meant for illustrative purpose. For both A and B, dots are color coded by lobe—frontal, purple; parietal, red; occipital, orange; temporal, cyan; and subcortical, green.

Having demonstrated that bilateral HTH gives the best seeding, we next captured the spatiotemporal evolution of ***x***^HTH^(*t*) (Figures 4A,B). The maximum of *R*^HTH^(*t*) occurs at *t*_max_ = 22, hence ***x***_**HTH**_(*t*_max_) is shown in Figure 4A. The ND progression matches quite closely the stereotypical sequencing in PSP, where the disease in due course extends into the subcortical and temporal structures and finally progresses to increasingly involve the cerebral cortex. The maximum correspondence of NDM to empirical data, given by the peak of the *R*–*t* curve in Figure 3B occurs at *t* = 22 for PSP. Figure 4B shows the evolution of network diffusion process seeded at the HTH starting at early stage (*t* = 7) through mature stage (*t* = 22), the model increasingly resembling empirical atrophy of Figure 1. We have selected three equidistant years at *t* = 7, 15, and 22 years between early stage (*t* = 0) with no diffusion through mature stage (*t* = 22). Here, time is arbitrary, and the use of “years” is meant for illustrative purpose. Initial spread is followed by diffusion especially into the pallidum and caudoputamen, followed by other striatal and limbic structures, then to middle temporal-frontal structures, and finally to the cortex.

### Testing for Significance against Alternate Randomized Models

**Figure 5:**
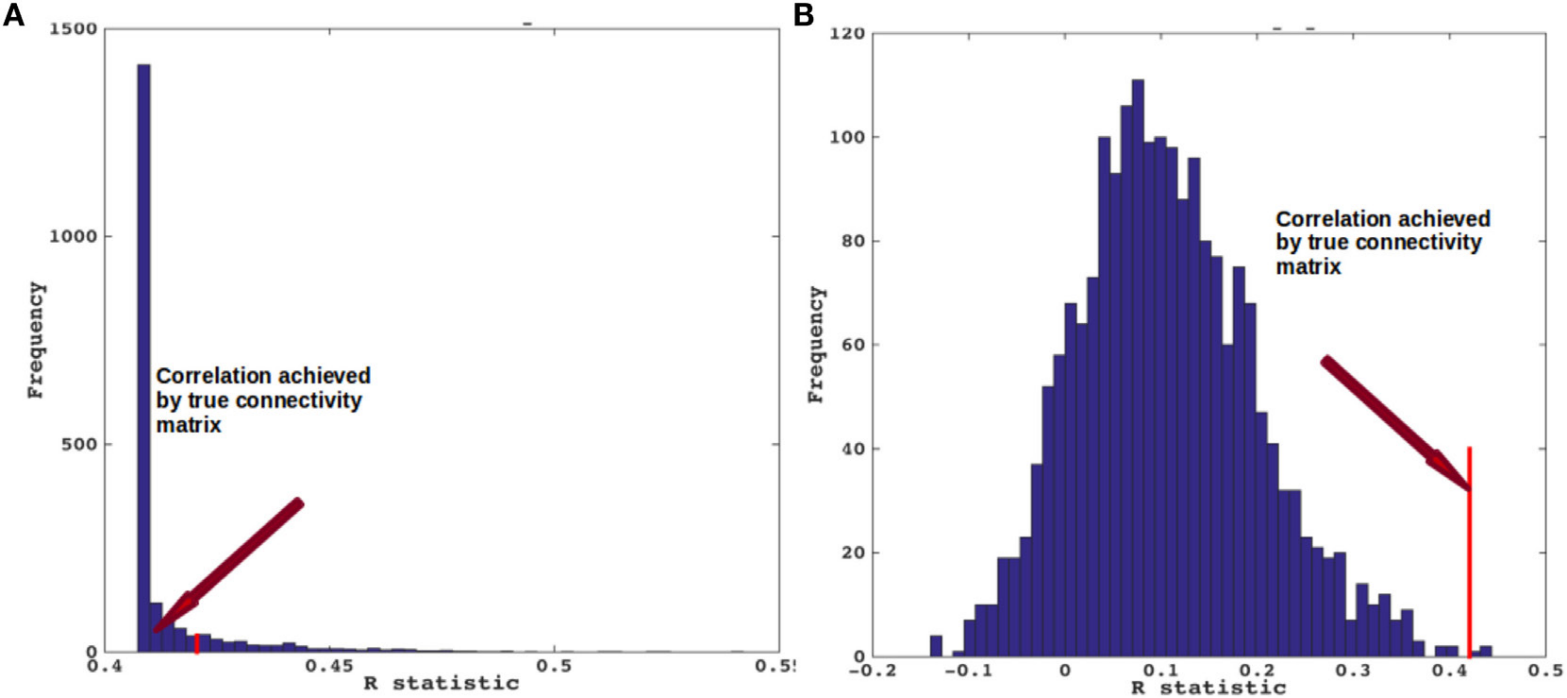
Scrambled networks and PSP atrophy. **(A)** Histogram of correlation strength between network diffusion model (NDM) and PSP data over 2,000 shuffled networks. **(B)** Histogram of correlation strength between NDM and 2,000 shuffled PSP data over using unshuffled structural connectome. The true connectome was shuffled by symmetrically permuting its rows and columns randomly, and the NDM was evaluated for each shuffled network after bilateral hypothalamus seeding. The best *R* achieved by each model was recorded and entered into the histogram. The null models are distributed well below the true model, indicating that the latter is highly unlikely to arise by chance (*p* < 0.05).

We evaluated the NDM against alternate network models to show its specificity to PSP atrophy and to the connectome on which it evolves. We evaluated this in two ways and recorded the best *R* achieved by each model. First, we randomly scrambled the healthy average connectivity matrix C 2,000 times, and ran the NDM on each scrambled network for PSP. Second, we randomly scrambled the group *t*-statistic of regional PSP atrophy vector and ran the NDM on original connectivity matrix C. The distribution of Pearson’s *R* over 2,000 scrambled matrices is shown in Figure 5A and clearly indicates that our correlation results are unlikely to be due to chance. There is a hard limit on the left of this plot at *R* ~ 0.42, which corresponds to the 0-diffusion time value of *R*^PSP^(*t*) curve in Figure 3B. The second random scrambling experiment, where the atrophy vector was scrambled instead of the connectome, gave an *R* distribution shown in Figure 5B. This distribution was approximately Gaussian, with mean between 0.1 and 0.2, and SD around 0.1 and 0.2. Random model’s *R* was much lower than the maximum *R* of 0.42 achieved by the true model; statistically outside the 95% confidence interval, or *p* < 0.05. Hence, the reported HTH seeded NDM prediction cannot be explained by chance.

### Repeated Seeding of the NDM with Directional Connectivity

**Figure 6:**
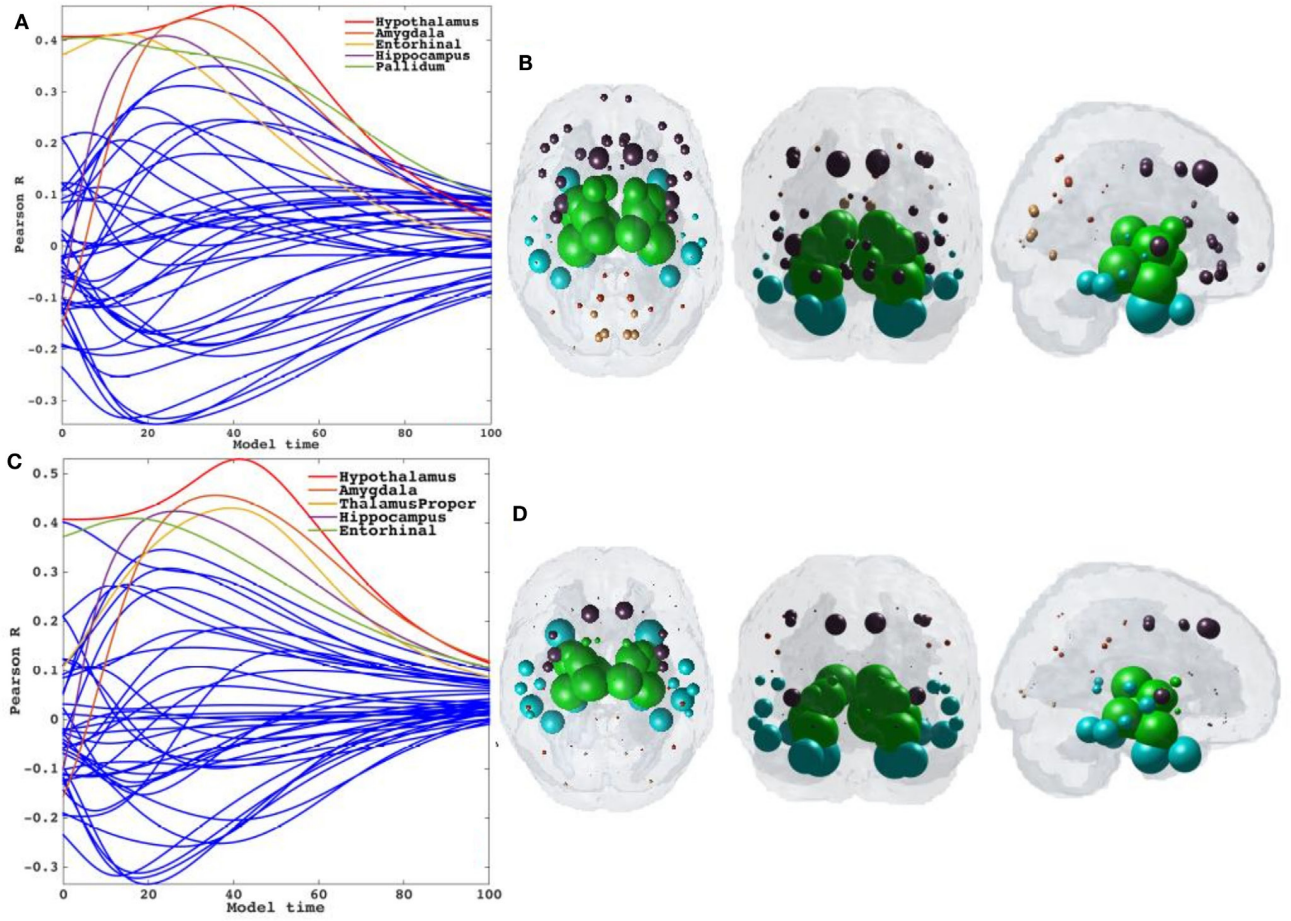
Results for repeated seeding analysis and ND prediction with directional connectivity. **(A,C)** Each region was seeded in turn and network diffusion model (NDM) was played out for all time points with directional connectivity. Pearson’s *R* was recorded at each time point between the model and PSP atrophy vector. As the diffusion time increases, more and more of the pathogenic agent escapes the seed region and enters the rest of the network. NDM seeded at bilateral regions using directional connectome indicates that the hypothalamus [shown by red curve in **(A)**] could be a plausible candidate for PSP disease seeding. The highest peak recorded with anterograde directional connectivity is *R* = 0.48 at *t* = 40 and with retrograde directional connectivity is *R* = 0.53 at *t* = 41. The characteristic intermediate peak is indicative of true pathology spread. The unit of model’s diffusion time *t* is arbitrary. For the purpose of demonstration, we consider this arbitrary timescale parameter in terms of “years.” **(B,D)** The point of maximum correlation *R* of NDM with measured atrophy using anterograde and retrograde directional connectome, respectively, were recorded and are shown with glass brains in **(B,D)**. For all subplots, dots are color coded by lobe—frontal, purple; parietal, red; occipital, orange; temporal, cyan; and subcortical, green.

Having demonstrated that bilateral HTH gives the best seeding, we next wanted to show if directionality could predict tauopathic spread either from same or different set of potential seeds. We tested both retrograde and anterograde mode in our model at *t* = 41 and *t* = 40, respectively. With anterograde connectivity there was an increase in maximum *R* (*R* = 0.48 at *t* = 40) as seen in *R*–*t* curve (Figure 6A). Our results showed that retrograde mode showed significant improvement in maximum *R* (*R* = 0.53 at *t* = 41) to both bidirectional and anterograde mode (Figure 6C). Both with retrograde and anterograde mode, the highest *R* was achieved by the HTH and served as the best seed region. Figures 6B,D show glass-brains with maximum *R* achieved by each region seeded in turn and Table 2 shows top 20 regions with maximum correlation strength for each region seeded in turn with directional connectivity.

### Directional Network Diffusion Recapitulates Regional Atrophy in PSP

**Figure 7:**
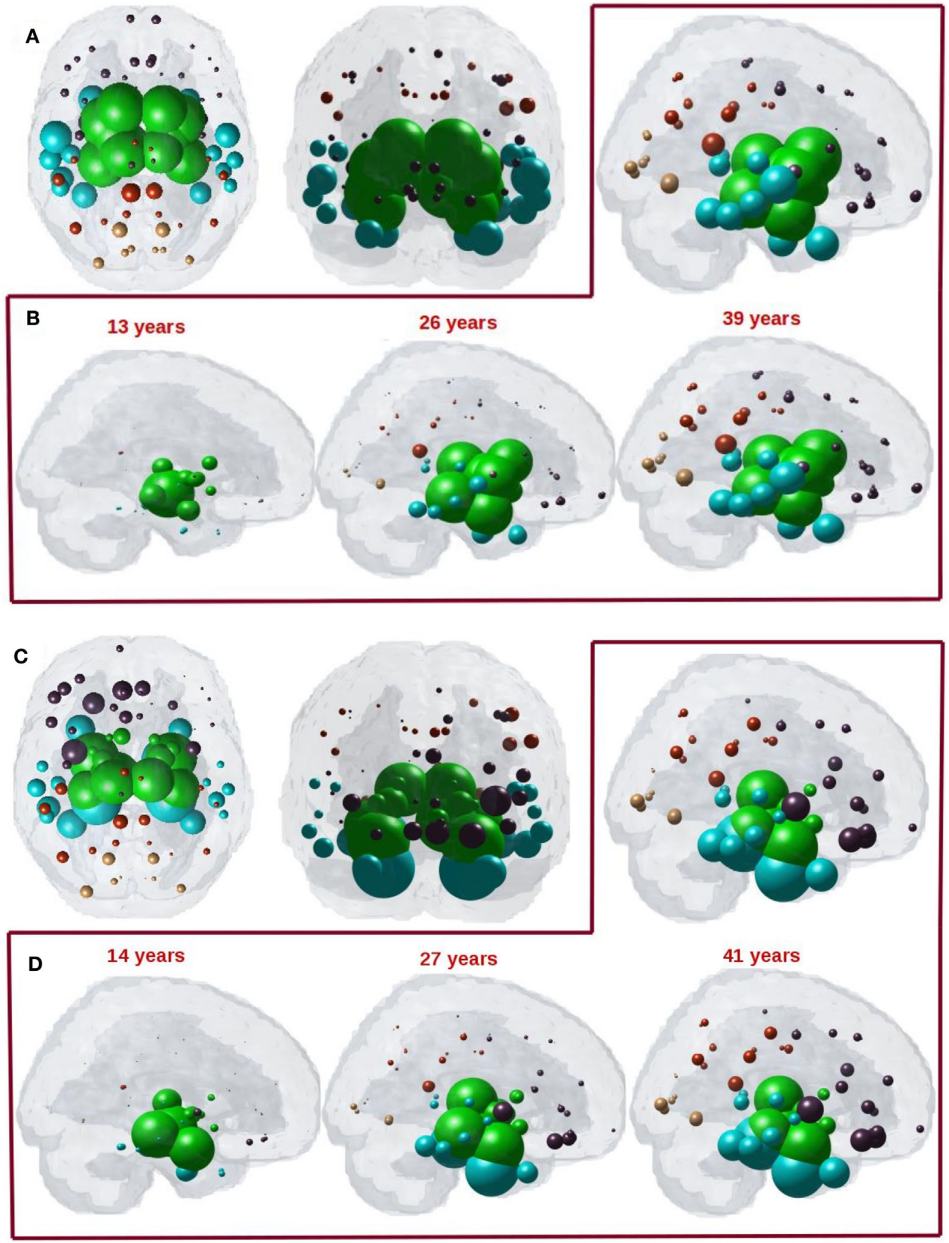
**(A)** ND prediction from bilaterally seeded hypothalamus (HTH) with anterograde directional connectome. Glass brains of network diffusion model (NDM) seeded at the bilateral HTH at *t* = 40 years (corresponding to highest peak *R*) shows spatial patterns of ND progression from HTH using anterograde connectivity. Similar to Figure 4A, even with anterograde directional connectivity the most affected regions are subcortical striatal structures especially caudoputamen and globus pallidus, and limbic areas like amygdala and hippocampus, which are shown in green. **(B)** Spatiotemporal evolution of empirical PSP pathology with anterograde directional connectivity. Evolution of HTH seeded network diffusion exhibits the, limbic, striatal, and temporal areas as early affected regions, followed by somewhat slower diffusion into the frontal and parietal regions. This spatial sequencing predicted by the model is a close match with PSP tau progression. We selected three equidistant years at *t* = 13, 26, and 39 years between early stage (*t* = 0) with no diffusion through mature stage (*t* = 39). Here, time is arbitrary, and the use of “years” is only meant for illustrative purpose. **(C)** ND prediction from bilaterally seeded HTH with retrograde directional connectome. Glass brains of NDM seeded at the bilateral HTH at *t* = 41 years (corresponding to highest peak *R*) shows spatial pattern from HTH using retrograde connectivity. With retrograde directional connectivity the next most affected region is entorhinal (big sphere in cyan) and then limbic areas like amygdala and hippocampus which are shown in green. **(D)** Spatiotemporal evolution of empirical PSP pathology with retrograde directional connectivity. Evolution of HTH seeded network diffusion exhibits the limbic and temporal areas as early affected regions, followed by somewhat slower diffusion into the striatal, frontal, and parietal regions. We selected three equidistant years at *t* = 14, 27, and 41 years between early stage (*t* = 0) with no diffusion through mature stage (*t* = 41). Here, time is arbitrary, and the use of “years” is only meant for illustrative purpose. For all subplots, dots are color coded by lobe—frontal, purple; parietal, red; occipital, orange; temporal, cyan; and subcortical, green.

We next captured the spatiotemporal evolution of ***x***^HTH^(*t*) (Figures 7A–D) at *t* = 40 and *t* = 41, which corresponds to time taken by diffusion to reach the highest peak with anterograde and retrograde directional connectivity, respectively. The maximum of *R*^HTH^(*t*) occurs at *t*_max_ = 40 and 41 for anterograde and retrograde mode, hence ***x***_**HTH**_(*t*_max_) are shown in Figures 7A,C for each mode, respectively. The ND progression shows better prediction of tau progression in PSP compared to non-directional seeding as seen in Figure 4. Both with anterograde and retrograde mode, the spreading pattern extends into the subcortical and temporal structures and finally progresses to increasingly involve the cerebral cortex.

The maximum correspondence of NDM to empirical data, given by the peak of the *R*–*t* curve in Figure 6A occurs at *R* = 0.48, *t* = 40 and in Figure 6C occurs at *R* = 0.53, *t* = 41 for PSP. Figures 7B,D shows the evolution of network diffusion process seeded at the HTH starting at early stage (*t* = 13) through mature stage (*t* = 39) with anterograde mode and through *t* = 14 and *t* = 41 with retrograde mode. With both the modes, the model increasingly resembles empirical atrophy of Figure 1 much so than with non-directional connectivity. We have selected three equidistant years at *t* = 13, 26, and 39 years between early stage (*t* = 0) with no diffusion through mature stage (*t* = 40) for anterograde mode and *t* = 14, 27, and 41 years between *t* = 0 and *t* = 41. Here, time is arbitrary, and the use of “years” is meant for illustrative purpose. As seen in Figure 7B with anterograde mode, initial seeding at HTH is followed by diffusion especially into the pallidum and caudoputamen, followed by limbic structures such as hippocampus and amygdala, then thalamus, then to middle temporal–frontal structures, and finally to the cortex. With retrograde mode (Figure 7D), initial seeding at HTH is followed by entorhinal, followed by other limbic structures such as hippocampus and amygdala, and thalamus, then to striatal, middle temporal-frontal structures, and finally to the cortex.

## Discussion

The current study applies network modeling to a rare disease as the first to systematically test hypotheses of disease spread in human subjects living with PSP. Other neurodegenerative diseases like FTD, AD, and ALS syndromes have been well-studied from a human brain network perspective, and have received more attention than equally important and related disorders (15), due to a lack of available human data on this rare disorder. The present analysis takes advantage of an unprecedented dataset of 60 subjects with PSP from a multinational prospective observation study called 4RTNI (see text footnote 1). Warren et al. proposed the term “molecular nexopathy” to refer to a coherent conjunction of pathogenic protein and intrinsic neural network characteristics (33). They enumerated a diverse set of potential mechanisms by which molecular dysfunction might interact with the neural architecture to produce observed disease topography in neurodegenerative diseases, including dysfunction of synaptic function or maintenance, axonal transport or repair, or a result of downstream trophic or cell–cell signaling. Although a full encapsulation of these various protein-specific mechanisms into a network model like ours will require much more detailed data and studies, here we have made a beginning by explicitly considering one of the key ways in which misfolded protein species might interact with the extant network—i.e., *via* directional (as compared to non-directional) transmission. We explored two mechanistic aspects hitherto unknown about the canonical NDM of spread: (a) whether the NDM can apply to other common degenerative pathologies, specifically PSP, and (b) whether the directionality of spread is important in explaining empirical data.

Our first key contribution is to show that the mathematical graph theoretic NDM of trans-neuronal transmission can apply to other degenerative pathologies, specifically it can recapitulate observed PSP topography. The interest in PSP arises from its distinct spatial pattern in comparison to AD; hence the ability of NDM to explain PSP pattern contributes to the emerging notion that all neurodegenerative pathologies follow shared mechanisms of spread. Our second key contribution is the novel construction of a *directional human connectome*, exploiting the homologies in brain anatomy that exist between species as diverse as human and mouse. The importance of studying directional connectomes is that *in vitro* and *in vivo* mouse studies are increasingly revealing directional preference in the transmission of various misfolded proteins; however, conclusive data on directionality of each protein are not currently established (24). Our third key contribution is to show that the NDM fit with empirical data are enhanced by using the directional rather than the non-directional form of the human connectome. Certain aspects of PSP topography are better explained by the anterograde model of transmission (some to axonal terminal) than the non-directional model, and certain aspects of PSP are better explained by the retrograde mode of transmission. Overall, both directional models outperform the non-directional model, and retrograde mode gives overall the best fit. These intriguing results are further discussed below.

### Focal Seeding Followed by Network Spread Recapitulates PSP Atrophy

We tested whether network spread from focal seed sites would recapitulate PSP regional atrophy patterns. Although trans-neuronal transmission appears the most likely candidate, the exact mode of transmission is unknown. We first simulated spread between regions in a bidirectional manner, such that pathology has an equal chance of spreading in the anterograde or retrograde direction. To minimize model bias, we performed repeated seeding such that each brain region in turn has a chance to serve as the single onset region from which subsequent pathology transmission was modeled using the NDM. The HTH was found to be the best seed region, with the highest model correspondence of *R* = 0.42. The model performance seeded at HTH is significant in comparison with random network scrambling of the structural connectome (Figure 5) (*p* < 0.05), confirming that network organization is integral to disease spread in PSP, rather than an effect that has arisen by chance. Surprisingly, it was a better seed than any region in the striatum, even though striatal atrophy is usually the most prominent feature of the topography of PSP. The next best seed region was Pallidum, which is certainly plausible as converging post mortem and neuroimaging studies showing the striatum is the most affected region of pathology in PSP (8, 34).

### Directional Structural Connectivity Model

Having identified structural connectivity as the most likely mechanism of transynaptic pathology spread in PSP, we sought to further improve our model by adding directionality. Clearly, a direct measurement of directionality of fiber tracts is impossible from current dMRI techniques, as water diffusion along fiber bundles does not respect cell polarity (soma to axonal terminal or *vice versa*). Instead, we exploit the well-known fact that homologous structures exist between many species, for some of whom we do happen to have anatomic connectivity data from painstaking tracer studies. A detailed mesoscale mouse connectome has recently become available from the Allen Institute (23). These data are fully directional, since it is based on retrograde AAV tracer experiments on a large number of mice. We, therefore, developed a novel technique whereby human and mouse brain atlas parcellations are used to define homologous brain structures, and mouse directional connectivity is transferred to non-directional human connectome.

Trans-neuronal transmission can have a distinct directional bias, such that misfolded protein species might follow anterograde or retrograde transport or signaling pathways. This is especially true in subcortical and striatal connections, which are known to be highly directional in comparison to corticocortical connections. Although little work has been done in PSP, tau pathology in AD differentiates between efferent and afferent connections (24). Hence, we tested the hypothesis that directional (whether anterograde or retrograde) process of spread along structural connectivity network will further improve model performance in PSP. The directional NDM results are shown in Figures 6 and 7, and confirm that either mode is a better fit than non-directional (Table 2). Interestingly, some aspects of PSP topography are better recapitulated with anterograde mode of transmission, especially striatal areas; whereas other aspects are better recapitulated by retrograde transmission, especially temporal and limbic areas. Quite unexpectedly, the HTH is the most likely source of pathology origin in all three modes of transmission, a result that raises many questions that should be addressed in future investigations.

## Conclusion

Collectively, these data show that the intrinsic architecture of the structural network mediates disease spread in PSP, most likely *via* a process of trans-neuronal transmission. The additional success of the directional network models suggests a simple process whereby local production of pathology starts off a process of axon-to-soma or soma-to-axon transmission, which due to the nature of the directional network topology, prominently accumulates in striatal and temporal areas. In this study, our focus was to build and apply directional NDMs in a purely data driven fashion, rather than a detailed exploration of the mechanistic aspects that govern directional transmission. We have shown that directionality of transmission may be an important aspect that network models should incorporate in future and related studies involving a wide range of neurodegenerative disorders. If confirmed by future, larger, studies, the apparent selective vulnerability and early seeding of PSP and other tauopathies in specific areas might plausibly entertain an explanation purely in terms of directional transmission, without requiring cell-type or region-specific vulnerability of brain region to pathology. To our mind, this would be the most parsimonious and economical reading of available data.

### Limitations

Several methodological considerations should be considered when interpreting our results. The first are limitations of the NDM. The NDM is a first-order, linear, parsimonious model of diffusive spread that assumes that the structural connectivity network remains unchanged over the duration of the longitudinal analysis. Moreover, individual subject genetic repeat length, medication history and age of symptom onset were not available. These variables could have implications in the individual group wise analysis, when identifying each subject’s seed region or determining individual rate of disease diffusion. Because this is the first study to empirically test multiple network models of pathology spread in PSP, it will benefit from independent replication. Future work elucidating striatal vulnerability as well as the effect of repeat length on disease spread is necessary.

## Author Contributions

SP coded, analyzed data, and wrote portions of the manuscript. CM extracted mouse and human connectome and wrote portions of manuscript. AR conceptualized the study, developed mathematical model, supervised image and statistical analysis, and wrote portions of the manuscript.

## Conflict of Interest Statement

The authors declare that the research was conducted in the absence of any commercial or financial relationships that could be construed as a potential conflict of interest.

## Footnotes

^1^http://4rtni-ftldni.ini.usc.edu.

^2^http://adni.loni.usc.edu.
